# A Cross-Sectional Study of Viral Hepatitis Perception among Residents from Southeast and North Regions of Brazil

**DOI:** 10.3390/ijerph15020189

**Published:** 2018-01-24

**Authors:** Helena Medina Cruz, Vanessa Salete de Paula, Livia Melo Villar

**Affiliations:** 1 Laboratory of Viral Hepatitis, Oswaldo Cruz Institute, FIOCRUZ, Rio de Janeiro 210360-040, Brazil; h.medina@ioc.fiocruz.br; 2 Molecular Virology Laboratory, Helio and Peggy Pereira Pavillion-Ground Floor, FIOCRUZ, Rio de Janeiro 210360-040, Brazil; vdepaula@ioc.fiocruz.br

**Keywords:** hepatitis, perception, urban population, Latin America

## Abstract

Few data are available regarding viral hepatitis perception among the general global population. The present study aims to estimate the perception of viral hepatitis in a cohort of individuals living in two geographical regions of Brazil: North (Manaus city (MA)) and Southeast (Rio de Janeiro city (RJ)). A cross-sectional, descriptive study was carried out among 287 subjects recruited in MA (134) and RJ (153). All individuals answered a questionnaire assessing socio-demographic characteristics and viral hepatitis awareness. Participants’ responses were scored and divided using interquartile values. Associations between socio-demographic characteristics and knowledge were also evaluated. Interquartile analysis scored 0–21 correct answers as “Very Weak”; 22–27 as “Weak”; 28–31 as “Intermediate”; and 32–47 as “Desirable”. Mean ± standard deviations (SD) of correct responses were weak in both MA (24.1 ± 7.0) and RJ (26.3 ± 7.3). Bivariate analysis showed an association between viral hepatitis awareness and both education level (*p* < 0.001) and family income (*p* < 0.01). Desirable scores were more common in female participants (61%), those aged between 21–30 years (40%), those with a secondary education (51.7%), those who received high income (31.6%), and those from RJ (70.0%). Health education campaigns in these cities are recommended to increase knowledge and reduce the transmission of these viruses.

## 1. Introduction

A group of viruses known as hepatitis A–E (HAV, HBV HCV, HDV, and HEV) cause viral hepatitis. HAV and HEV are transmitted by ingestion of contaminated food or water while HBV, HCV, and HDV are usually transmitted as a result of parenteral contact with infected bodily fluids, such as during transfusion of contaminated blood or blood products, invasive medical procedures using contaminated equipment, sexual intercourse, and horizontal and vertical transmission [1,2,3].

Viral hepatitis is the eighth primary cause of mortality worldwide, resulting in 1.44 million deaths in 2010 [2]. In Brazil, the prevalences of HAV, HBV, and HCV are low [4,5,6]. The incidence of cases per 100,000 inhabitants in 2016 of HAV, HBV, and HCV were 0.7, 14.0, and 10.8, respectively, in Manaus (MA) and 0.3, 3.3, and 13.4, respectively, in Rio de Janeiro (RJ) [4]. The confirmed cases of HAV, HBV, HCV, and HDV represent 25.8%, 14.2%, 3.1% and 76.8% of all cases in the North region of Brazil, respectively, and 16.4%, 35.4%, 62.2% and 9.8% in the Southern areas of Brazil, respectively, in 2016. Regarding HEV infection, Brazil is considered a moderate endemicity region where HEV prevalence varies from 1% in pregnant women to 17.7% in women at risk for HIV [7].

According to the United Nations, by 2030, their agenda intends to finish the epidemics of AIDS, tuberculosis, malaria, and neglected tropical diseases and to also combat hepatitis, waterborne diseases and other communicable diseases. According to global objectives, it is fundamental to promote universal access to information and education in order to prevent infectious diseases like hepatitis [8]. To this end, in order to plan preventive measures for viral hepatitis, it is essential to identify the gaps in viral hepatitis perception in the general population.

Most infected individuals may not present clinical manifestations, or these manifestations may appear years after infection when the disease is already in an advanced stage [1,3]. The most common clinical manifestations of viral hepatitis are fever; weakness; abdominal pain; sickness, nausea; vomiting; loss of appetite; dark urine; jaundice; and pale feces [1]. The progression of HBV and HCV infection could lead to cirrhosis and hepatocellular carcinoma (HCC) [9].

Laboratory diagnosis of viral hepatitis includes enzyme immunoassays (EIA) or electrochemiluminescence immunoassays (ECLIA) to detect specific viral hepatitis antigens or antibodies, and molecular assays such as polymerase chain reaction (PCR) to detect and quantify the viral genome [10]. Currently, safe and effective vaccines are available for hepatitis A and B prevention, but there are no vaccines for other forms of viral hepatitis [2].

Some studies have shown that awareness of hepatitis can be influenced by many factors, including education, health literacy, family income, age, the knowledge of the severity of illness, and access to information [11,12]. Viral hepatitis perception also varies according to occupation and individual characteristics. In Iran, hairdressers with secondary school education demonstrated a high level of knowledge about HBV and HCV [10]. An inadequate knowledge score was obtained by health professionals and students in Ethiopia [13] and, in studies performed in China and the USA, knowledge scores were related to study site, education, gender, and prior HCV treatment [12].

Few studies have been done to identify perception regarding the five hepatitis viruses in the general population. A poor perception was observed among the general population in Pakistan [14] and Vietnamese Americans in the USA [15]; however, to our knowledge, there is no study regarding the understanding of viral hepatitis in the general population of Brazil. The present study aims to estimate the perception about viral hepatitis A, B, C, D, and E in a cohort of individuals from general populations living in two cities of North and Southern Brazil, to identify possible gaps and strengths.

## 2. Methods

### 2.1. Study Population

A cross-sectional, descriptive study was carried out to determine viral hepatitis perception among Brazilian individuals living in two cities from North and Southeast Brazil (MA and RJ, respectively). Interviews were conducted in RJ in June 2009 and in MA from July to August 2009 during public health campaigns for infectious disease prevention, such as poliovirus vaccination. All individuals were from the metropolitan region of Rio de Janeiro and North and South–Central regions of Manaus city.

RJ city is situated in the Southern region of Brazil and has 6,453,682 residents, while MA city is located in the Northern region of the country and has 2,020,301 residents. RJ and MA are the second and the seventh most populous cities in Brazil, respectively [16].

The group of participants comprised both genders and inclusion criteria required participants to be 18 years of age and provide signed, informed consent. A sample size of 100 individuals for each location was targeted. Assuming a response rate of 75–80%, 75 completed questionnaires would yield a power of 80% with a 5% type 1 error rate to detect a 16% difference when comparing dichotomous variables between two groups of equal size. The final sample was made up of 287 individuals. No incentive was given to these individuals to participate in this study. The local ethical committee approved the study (protocol 0086.0.317.000-08).

### 2.2. Questionnaire

A specific questionnaire was developed to determine viral hepatitis perception. This instrument was composed of two topics: demographic characteristics and viral hepatitis perception.

Sociodemographic data included gender, age, education, and monthly family income. Monthly family income was determined according to the Brazilian minimum salary (US $276.00). In this fashion, individuals who receive <US $276.00 were grouped as “low family income”, individuals who received US $276.00 to US $828.00 were grouped as “intermediate family income”, and individuals who receive more than US $828.00 were grouped as “high family income”.

**Figure 1:**
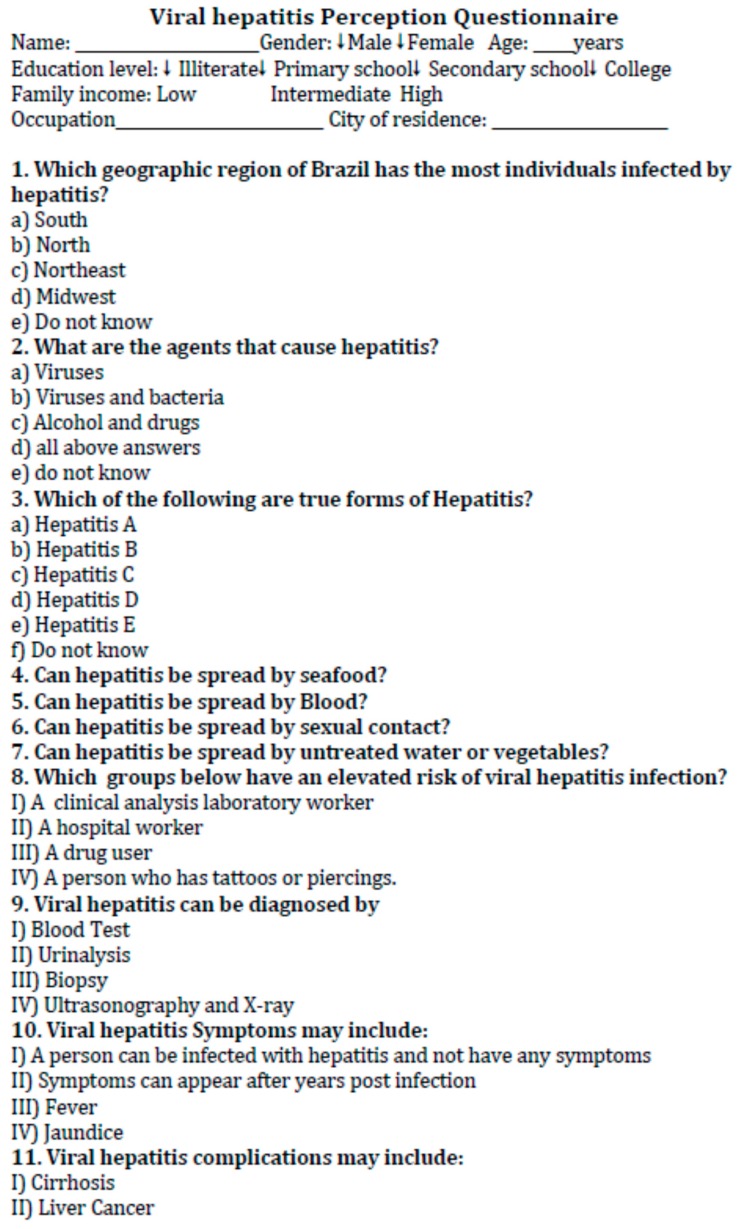

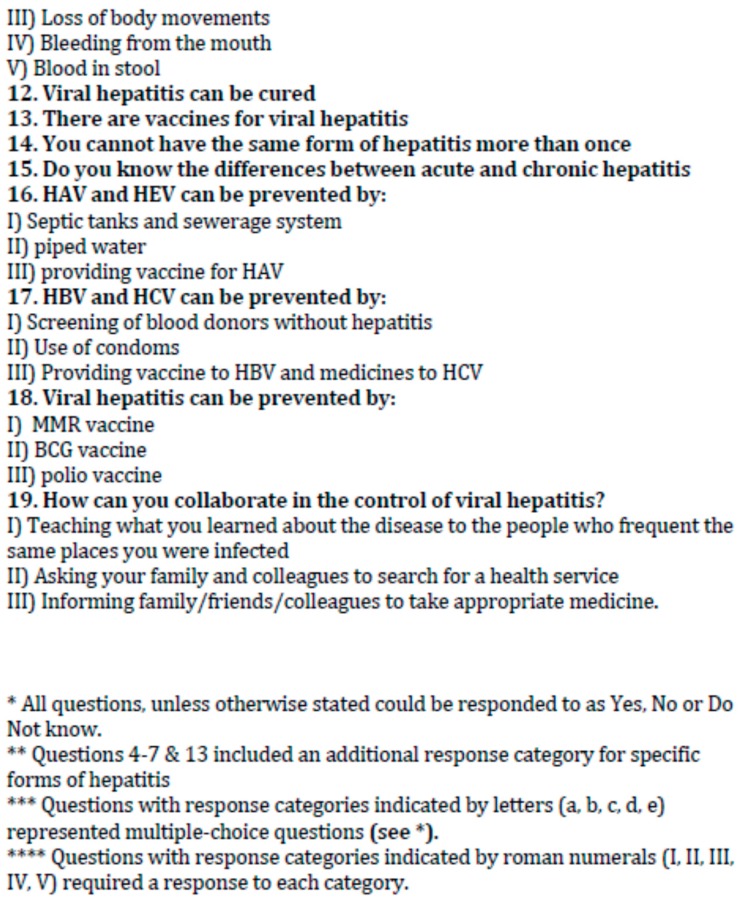
Questionnaire used to evaluate Viral Hepatitis Perception. Legend: HAV: Hepatitis A virus; HBV: Hepatitis B virus, HCV: Hepatitis C virus; HDV: Hepatitis D virus; HEV: Hepatitis E virus; MMR: Measles, mumps, and rubella; BCG: Bacille Calmette-Guerin.

Participants’ understanding of some viral hepatitis aspects, such as general information, diagnosis, clinical manifestation, transmission, risk factors, complications, and prevention, were assessed. This section of the questionnaire consisted of 3 questions requiring one or more responses and a further 16 questions with the following options: “yes/correct”, “no/incorrect”, or “do not know”. In five questions, individuals were required to inform the type of hepatitis viruses related to their responses (items 4–7, 13) (Figure 1).

The authors developed this questionnaire following a review of the literature on viral hepatitis aspects [1]. A total of 47 correct answers could be achieved. The questionnaire was standardized by applying it to a set of individuals characteristically representative of but not included in the study population (data not shown and statistical analysis not conducted). Interviewers administrated the questionnaire to the participants as a face-to-face interview in a confidential setting. At the end of the interview, the correct answers were shown to each volunteer. Authors asked for participants in the recruitment setting not to reveal the answers of this interview to other potential participants.

Descriptive statistics were generated for the responses and a chi-squared test for independence or trend was used to compare categorical and continuous variables, respectively, among the perception score groups. The Kruskal–Wallis test was used to evaluate the relationship between nominal and ordinal variables and a *p*-value < 0.05 was considered statistically significant. A viral hepatitis perception score was created based on all participants’ responses according to interquartile values. The 1–4 quartiles were considered very weak, weak, intermediate, and desirable, respectively. Associations between socio-demographic characteristics and perception were also evaluated. All analyses were performed using the Statistical Package for the Social Sciences (SPSS for Windows, release 20.0, SPSS, Chicago, IL, USA).

## 3. Results

### 3.1. Demographic Characteristics

A total of 287 individuals were recruited—134 from MA and 153 from RJ. Most participants were female, had a secondary education, were aged between 21–30 years, and had a low monthly family income. Main socio-demographic characteristics are shown in Table 1.

### 3.2. Viral Hepatitis Perception in Manaus City

In MA city, most respondents (>70%) were aware of the existence of hepatitis A–C. However, more than 50% considered hepatitis D and E to be nonexistent. Furthermore, most individuals were unaware that hepatitis can be cured, that individuals cannot have the same form of hepatitis more than once, that there are vaccines for viral hepatitis, and of the differences between acute and chronic hepatitis (Table 2).

Most of the individuals declared that they could collaborate to control hepatitis by “teaching what you learned about the disease to the people who frequent the same places you were infected” (96.3%) and “informing family and colleagues to search for a health service” (93.3%) (Table 2). It is important to recognize that the information about viral hepatitis should be transmitted to other individuals to help in prevention and control.

Most of the participants claimed that a blood test can be used for diagnosis (91.8%) and that jaundice and fever are symptoms of hepatitis infection (91.0% and 76.1%, respectively) (Table 2). Regarding transmission, most participants responded that blood (79.9%), sexual contact (70.1%), and water or vegetables without treatment could transmit hepatitis (62.7%), but a minority of participants recognized seafood (26.1%) as a vehicle of transmission. Also, most participants were unable to identify precisely the virus transmitted by seafood, blood, sexual intercourse, or ingestion of untreated water or vegetables (percentages varied from 76.9 to 91.8%) (Table 3).

A majority of respondents recognized healthcare workers in hospitals and clinical analysis laboratories as groups at risk of acquiring hepatitis (87.3% and 88.1%, respectively). Additionally, most individuals were able to identify cirrhosis (85.8%) and cancer (79.9%) as complications of hepatitis infection (Table 3).

Regarding hepatitis prevention, more than 78% of individuals affirmed that septic tanks, sewage systems, piped water, screening of blood donors, and the use of condoms are means of transmission prevention (Table 3). Half of the participants cited the existence of viral hepatitis vaccines, but 85% of them did not know the forms of hepatitis against which the vaccines were active (Table 2) and more than 70% claimed that the measles, mumps, and rubella (MMR) vaccine can prevent viral hepatitis (Table 3).

### 3.3. Viral Hepatitis Perception in Rio de Janeiro City

A majority of individuals from RJ (>74%) knew of the existence of hepatitis A–C. Also, most individuals (60.8%) affirmed that hepatitis could be cured and more than 67% did not believe that hepatitis D and E exist (Table 2).

Most of the participants declared that the population could collaborate to control hepatitis by “teaching what you learned about the disease to the people who frequent the same places you were infected” (78.4%), and “informing family and colleagues to search for a health service” (83.0%) (Table 2).

Most individuals cited blood tests (95.4%) and biopsies (65.5%) as diagnostic tools for viral hepatitis and more than 65% of individuals recognized jaundice (76.5%) and fever (66.0%) as clinical manifestations of infection (Table 2). Most participants also recognized blood (88.2%), untreated water or vegetables (83.7%), and sexual intercourse (75.8%) as vehicles for hepatitis transmission, and less than 40% of individuals identified seafood as a mode of transmission. Additionally, most of the participants did not know precisely the virus transmitted by seafood, blood, sexual intercourse, or untreated water or vegetables (Table 3).

A majority of individuals cited individuals working in clinical analysis laboratories (85.0%) or hospitals (78.4%), those with tattoos or piercings (76.5%), and drug users (71.9%) as those more vulnerable to hepatitis infection. Cirrhosis (68%) and cancer (58.2) were identified as hepatitis complications (Table 3).

Most of the participants cited the use of condoms (73.9%), screening of blood donors (70.6%), piped water (69.3%), and septic tanks and sewage systems (64.4%) as effective measures in the prevention of hepatitis transmission (Table 3). Among the participants, 72.5%% were aware of viral hepatitis vaccines, but 75.8% did not know the type of virus prevented by vaccination (Table 2) and 46.4% of individuals believed that the MMR vaccine can prevent viral hepatitis (Table 3).

### 3.4. Perception about Viral Hepatitis According to Demographic Characteristics

The interquartile analysis produced knowledge scores as follows: “Very Weak” (0–21 correct answers); “Weak” (22–27 answers); “Intermediate” (28–31 answers); and “Desirable” (32–47 answers). The Average score of correct answers from all participants was 25.3 ± 7.2 (24.1 ± 7.0 from MA city and 26.3 ± 7.3 from RJ city), which was considered to be weak according to interquartile analysis.

According to correct answers, 70 (52.2%) individuals in MA and 87 (56.9%) in RJ scored above the respective mean scores. Also, the percentage of individuals in the scores “very weak”, “weak”, “intermediate” and “desirable”, respectively, were 30.6%, 35.1%, 20.9%, and 8.7% in MA and 20.3%, 25.5%, 26.8%, and 27.4% in RJ.

Females and individuals aged between 31–40 years presented a high mean of correct answers, although it was not significant. Perception was associated with education (*p* < 0.001), family income (*p* = 0.001), and city of residence (*p* = 0.001) in bivariate analysis (Table 4).

Desirable scores were more common among females (61%), subjects aged between 21–30 years (40%), those presenting secondary school education (51.7%), and residents from RJ (70.0%). “Very weak” and “weak” perception was more common among individuals who had low family income (71.2% and 85.3%, respectively) (Table 4).

## 4. Discussion

In this study, a poor public perception regarding viral hepatitis was observed in North and Southeast regions of Brazil (average score of 25.3 ± 7.2 correct answers), and knowledge level was significantly associated with family income, level of education, and city of residence.

Most of the individuals answered more than 50% of the questions correctly, but some wrong answers occurred in all sections of the questionnaire (general information, diagnosis, clinical manifestations, transmission, risk factors, complications, and prevention). Saleh et al. [17] found that absence of knowledge among different groups and levels of literacy is not unique to HCV but can also be observed for other forms of hepatitis.

Although the subjects recognized the existence of viral hepatitis, they were unable to identify which hepatitis viruses are transmitted by seafood, blood, sexual contact, or water or vegetables without treatment. Strong et al. (2015) [14] affirmed that misunderstanding of the transmission route may strengthen the stigma against individuals with HBV. The inability to recognize the transmission pathways of different viruses can influence the prevention of these diseases.

Several individuals did not recognize the existence of hepatitis D and E, claimed that blood in the stool can be a complication of viral hepatitis, and did not know that a biopsy could be used for diagnosis. Also, more than half of subjects from both cities did not recognize the differences between the acute and chronic forms of the disease and did not know that the same form of hepatitis cannot be acquired more than once. The difficulty participants had with some general questions about hepatitis demonstrates the importance of health education programs to increase viral hepatitis awareness.

Low knowledge of HEV could be related to the absence of routine, specific HEV diagnosis in central laboratories [7]. This situation could lead to potential cases being unreported. Additionally, while vertical transmission is less common, the mortality of children born to HEV-infected mothers is high [18]. As such, both under-reporting and a lack of prenatal testing of HEV pose both a threat to those at risk and an under-checked source of transmission.

Some questions of this survey could have had more than one answer, such as “can viral hepatitis be cured?”. In HBV and HDV infections, there is no cure for chronic infections. This imprecision may have influenced the evaluation of the knowledge of this question; however, as mentioned previously, most of the participants did not recognize the differences between the acute and chronic form and this unfamiliarity could also influence this evaluation.

The majority of respondents recognized the vehicles of transmission as the same as those observed by hairdressers regarding HCV transmission [19]. On the other hand, 81% of participants from a rural village of Egypt did not recognize the routes of HCV transmission and 38% of barbers from Pakistan did not know the transmission routes of HBV and HCV [17,20,21].

In the present study, seafood was not recognized as a source of viral hepatitis transmission, although ingestion of seafood can transmit HAV and HEV [22,23]. This situation could increase the transmission of viruses through this route if individuals are not cognizant of cleaning and disinfection of seafood as viable means of reducing their vulnerability to infection.

Most of the individuals recognized that viral hepatitis diagnosis is made using blood samples, but in MA city, many subjects did not identify biopsies as a method for hepatitis diagnosis. Biopsies are an additional method that allow the prediction of the evolution of the disease. In MA city almost half of the participants reported that urine could diagnose viral hepatitis. Viral hepatitis markers are detected in urine and other biological fluids such as saliva, semen, breast milk, pancreatic secretions, bile and vaginal, tears, menstrual, pleural, and nasopharyngeal [9,24], but the efficiency of detection in fluids like urine is not ideal for diagnosis of all types of hepatitis viruses [25,26,27].

More than 50% of individuals from both cities recognized clinical manifestations of viral hepatitis, but many of them did not know that these symptoms could appear years after the establishment of infection or may not manifest at all. Saleh et al. [17] showed that 45% of the participants did not know about the clinical manifestations of HCV.

The existence of a viral hepatitis vaccine was correctly reported by 50.0% of subjects from MA and 72.5% from RJ. However, 85.1% of respondents from MA and 75.8% from RJ did not know whether or not there were vaccines for HAV and HBV. Unfamiliarity with the existence of HAV and HBV vaccines is an alarming situation since vaccines for HAV and HBV are available in Brazilian Immunization Programs, and would reduce the burden of these infections in our setting. The HAV vaccine was included in the National Immunization Program and has been available in public health clinics for children under two years of age since 2014. HBV vaccination began in 1989 in the Brazilian Amazon region. In 1992, it was offered to all Brazilian children under two years of age and since 1998 it has been recommended in Brazil for all children at birth.

Among the confirmed cases of HDV in Brazil from 1999 to 2016, 78.6% occurred in the North region, and, among these, 50.8% in the Amazonas state [4]. Although the individuals interviewed in MA belong to this state and region, almost 60% of the participants did not know about HDV. These findings, together with the low knowledge about the HBV vaccine—which is a form of HDV prevention—demonstrates a lack of access to information in the North region about this infection and the need for better knowledge regarding measures of HDV prevention.

Viral hepatitis perception was associated with education level and monthly family income. A desirable perception was observed among those who have at least completed secondary school and very weak perception among individuals with low family income. Amodio et al. [19] also saw low awareness of viral hepatitis in hairdressers with low education in Italy. These results demonstrate the role of formal education in the dissemination of infectious disease perception and the absence of knowledge in poor communities.

The present study has some limitations: (i) absence of information regarding the neighborhood of each participant; (ii) high diversity of occupations reported by each in this sample; (iii) lack of control for confounding factors and multivariate analysis model; (iv) the study was conducted in 2009 and several measures by public health authorities in Brazil have since been conducted. These measures included the publication of clinical guidelines for therapy of Hepatitis B and C, Brazilian seroprevalence studies for Hepatitis A–C, the inclusion of rapid tests for diagnosis, the distribution of HBV vaccines to all individuals, and the inclusion of new direct antiviral agents for HCV treatment. Despite these limitations, few data are available regarding the knowledge of hepatitis and the present study is important in showing the concepts about these viruses in this period. The data observed is pertinent to the improvement of methods for assessing the public knowledge of viral hepatitis.

## 5. Conclusions

In conclusion, a weak level of perception about viral hepatitis was observed in both cities. Knowledge levels were associated with education and family income. It is necessary to conduct health education campaigns to increase viral hepatitis awareness in these cities so that the transmission of these viruses might be reduced.

## Figures and Tables

**Table 1:** Socio-demographic characteristics of participants.

Items	Total (287)
	*n* (%)
Local	
Rio de Janeiro	153 (53.3)
Manaus	134 (46.7)
Gender	
Female	173 (60.3)
Male	114 (39.7)
Age groups (years)	
18–21	34 (11.8)
21–30	115 (40.1)
31–40	66 (23.0)
41–50	41 (14.3)
>50	29 (10.1)
Not declared	2 (0.7)
Education	
Illiterate	26 (9.1)
Primary school	42 (14.6)
Secondary school	162 (56.4)
College	56 (19.5)
Not declared	1 (0.4)
Family income	
Low	157 (54.7)
Intermediate	57 (19.9)
High	42 (14.6)
Not declared	31 (10.8)

**Table 2:** Knowledge about general aspects, diagnosis, and symptoms of viral hepatitis among Manaus (MA) and Rio de Janeiro (RJ) participants.

Statement	Number (%)					
	Manaus (*n* = 134)			Rio de Janeiro (*n* = 153)		
	Correct	Incorrect	Do Not Know	Correct	Incorrect	Do Not Know
General information						
There is hepatitis A	94 (70.1)	11 (8.2)	29 (21.6)	120 (78.4)	33 (21.6)	0 (0)
There is hepatitis B	94 (70.1)	11 (8.2)	29 (21.6)	132 (86.3)	21 (13.7)	0 (0.0)
There is hepatitis C	94 (70.1)	11 (8.2)	29 (21.6)	114 (74.5)	39 (25.5)	0 (0.0)
There is hepatitis D	56 (41.8)	49 (36.6)	29 (21.6)	41 (26.8)	112 (73.2)	0 (0.0)
There is hepatitis E	48 (35.8)	57 (42.6)	29 (21.6)	50 (32.7)	103 (67.3)	0 (0.0)
Viral hepatitis can be cured	44 (32.8)	34 (25.4)	56 (41.8)	93 (60.8)	28 (18.3)	32 (20.9)
There are vaccines for viral hepatitis	67 (50.0)	18 (13.4)	49 (36.6)	111 (72.5)	6 (3.9)	36 (23.5)
There are vaccines for HAV and HBV	16 (11.9)	4 (3.0)	114 (85.1)	25 (16.4)	12 (7.8)	116 (75.8)
You cannot have the same hepatitis more than once	50 (37.3)	22 (16.4)	62 (46.3)	62 (40.5)	39 (25.5)	52 (34.0)
There are differences between acute and chronic hepatitis	22 (16.4)	112 (83.6)	0 (0.0)	47 (30.7)	104 (68.0)	2 (1.3)
You can help to control hepatitis by teaching what you have learned to other individuals who frequent the same place where you were infected	129 (96.3)	2 (1.5)	3 (2.2)	120 (78.4)	15 (9.8)	18 (11.8)
You can help to control hepatitis by informing family and colleagues to search for a health service	125 (93.3)	5 (3.7)	4 (3.0)	127 (83.0)	8 (5.2)	18 (11.8)
You can help to control hepatitis by informing family and colleagues to buy and take appropriate medicine to inactivate the virus	66 (49.3)	46 (34.3)	22 (16.4)	84 (54.9)	51 (33.3)	18 (11.8)
Diagnosis						
Hepatitis can be diagnosed by blood test	123 (91.8)	5 (3.7)	6 (4.5)	146 (95.4)	6 (3.9)	1 (0.7)
Hepatitis cannot be diagnosed by Urinalysis	59 (44.0)	59 (44.0)	16 (11.9)	110 (71.9)	41 (26.8)	2 (1.3)
Hepatitis can be diagnosed by Biopsy	24 (17.9)	94 (70.1)	16 (11.9)	100 (65.4)	50 (32.7)	3 (2.0)
Hepatitis cannot be diagnosed by X-ray	20 (14.9)	91 (67.9)	23 (17.2)	140 (91.5)	12 (7.8)	1 (0.7)
Symptoms						
Absence of symptoms	64 (47.8)	54 (40.3)	16 (11.9)	86 (56.2)	21 (13.7)	46 (30.1)
Symptoms can appear years after infection	62 (46.3)	55 (41.0)	17 (12.7)	62 (40.5)	69 (45.1)	22 (14.4)
Fever can be a symptom	102 (76.1)	17 (12.7)	15 (11.2)	101 (66.0)	30 (19.6)	22 (14.4)
Jaundice can be a symptom	122 (91.0)	2 (1.5)	10 (7.5)	117 (76.5)	15 (9.8)	21 (13.7)

**Table 3:** Knowledge about transmission, risks, prevention, and complications of viral hepatitis among MA and RJ participants.

Questions	Number (%)					
	Manaus (*n* = 134)			Rio de Janeiro (*n* = 153)		
	Correct	Incorrect	Do Not Know	Correct	Incorrect	Do Not Know
Hepatitis can be spread by						
Seafood	35 (26.1)	96 (71.6)	3 (2.2)	61 (39.9)	74 (48.4)	18 (11.8)
HAV and HEV can be transmitted by seafood	7 (5.2)	4 (3.0)	123 (91.8)	3 (2.0)	3 (2.0)	147 (96.0)
Blood	107 (79.9)	27 (20.1)	0 (0.0)	135 (88.2)	14 (9.2)	4 (2.6)
HBV, HCV, and HDV can be transmitted by blood	18 (13.4)	13 (9.7)	103 (76.9)	24 (15.7)	7 (4.6)	122 (79.7)
Sexual contact	94 (70.1)	39 (29.1)	1 (0.7)	116 (75.8)	30 (19.6)	7 (4.6)
HBV, HCV, and HDV can be transmitted by sexual contact	19 (14.2)	10 (7.5)	105 (78.3)	23 (15.0)	4 (2.6)	126 (82.4)
Water or vegetables without treatment	84 (62.7)	50 (37.3)	0 (0.0)	128 (83.7)	17 (11.1)	7 (4.6)
HAV and HEV can be transmitted by water or vegetables without treatment	12 (8.9)	6 (4.5)	116 (86.6)	17 (11.1)	10 (6.5)	126 (82.4)
People at risk of acquiring hepatitis						
Drug users	63 (47.0)	71 (53.0)	0 (0.0)	130 (85.0)	18 (11.8)	5 (3.3)
People with tattoos or piercings	67 (50.0)	67 (50.0)	0 (0.0)	120 (78.4)	28 (18.3)	5 (3.3)
Hospital Employees	117 (87.3)	17 (12.7)	0 (0.0)	117 (76.5)	31 (20.3)	5 (3.3)
Clinical laboratory workers	118 (88.1)	16 (11.9)	0 (0.0)	110 (71.9)	38 (24.8)	5 (3.3)
Complications						
Hepatitis can lead to cirrhosis	115 (85.8)	3 (2.2)	16 (11.9)	104 (68.0)	19 (12.4)	30 (19.6)
Hepatitis can lead to liver cancer	107 (79.9)	15 (11.2)	12 (9.0)	89 (58.2)	33 (21.6)	31 (20.3)
Hepatitis cannot lead to loss of body movements	68 (50.7)	23 (17.2)	43 (32.1)	83 (54.2)	38 (24.8)	32 (20.9)
Hepatitis cannot lead to bleeding from mouth	33 (24.6)	66 (49.3)	35 (26.1)	78 (51.0)	45 (29.4)	30 (19.6)
Hepatitis cannot lead to blood in stool	25 (18.7)	65 (48.5)	44 (32.8)	62 (40.5)	61 (39.9)	30 (19.6)
Prevention						
HAV and HEV can be prevented by septic tanks and sewerage systems	116 (86.6)	6 (4.5)	12 (9.0)	100 (65.4)	25 (16.3)	28 (18.3)
HAV and HEV can be prevented by piped water	114 (85.1)	9 (6.7)	11 (8.2)	106 (69.3)	19 (12.4)	28 (18.3)
HAV and HEV can be prevented by providing vaccine for HAV	108 (80.6)	15 (11.2)	11 (8.2)	97 (63.4)	28 (18.3)	28 (18.3)
HBV and HCV can be prevented by selecting blood donors not infected by hepatitis	111 (82.8)	7 (5.2)	16 (11.9)	108 (70.6)	20 (13.1)	25 (16.3)
HBV and HCV can be prevented by use of condoms	105 (78.4)	10 (7.5)	19 (14.2)	113 (73.9)	16 (10.5)	24 (15.7)
HBV and HCV can be prevented by providing vaccine and drugs	104 (77.6)	9 (6.7)	21 (15.7)	118 (77.1)	10 (6.5)	25 (16.3)
Vaccine to measles, mumps, and rubella (MMR) cannot prevent hepatitis	39 (29.1)	58 (43.3)	37 (27.6)	59 (38.6)	71 (46.4)	23 (15.0)
Vaccine to BCG cannot prevent hepatitis	67 (50.0)	24 (17.9)	43 (32.1)	110 (71.9)	18 (11.8)	25 (16.3)
Vaccine to POLIO cannot prevent hepatitis	79 (59.0)	17 (12.7)	38 (28.4)	115 (75.2)	15 (9.8)	23 (15.0)

Legend: HAV: Hepatitis A virus, HBV: Hepatitis B virus, HCV: Hepatitis C virus, HEV: Hepatitis E virus.

**Table 4:** Socio-demographic characteristics according to knowledge scores for viral hepatitis in the population studied.

Item	Mean Score (SD)	Knowledge Levels * *n* (%)				*p*-Value
		Very Weak	Weak	Intermediate	Desirable	
Gender						
Male	25.1 (7.7)	31 (43.05)	33 (38.4)	27 (39.1)	23 (38.3)	0.617
Female	25.4 (6.9)	41 (56.95)	53 (61.6)	42(60.9)	37 (61.7)	
Age group (years)						
≤20	23.7 (8.0)	13 (18.1)	7 (8.1)	8 (11.6)	6 (10.0)	0.673
21–30	25.0 (7.5)	24 (33.3)	46 (53.5)	23 (33.3)	24 (40.0)	
31–40	27.2 (6.4)	12 (16.6)	16 (18.6)	17 (24.6)	21 (35.0)	
41–50	25.0 (7.1)	13 (18.1)	8 (9.3)	12 (17.4)	8 (13.3)	
≥51	24.0 (6.0)	10 (13.9)	9 (10.5)	9 (13.1)	1 (1.7)	
Education						
Illiterate	23.3 (7.0)	8 (11.1)	10 (11.8)	8 (11.6)	0 (0.0)	<0.001
Primary School	22.9 (7.2)	13 (18.1)	15 (17.6)	11 (15.9)	3 (5.0)	
Secondary School	24.8 (7.2)	45 (62.5)	50 (58.8)	36 (52.2)	31 (51.7)	
Graduated	29.1 (6.0)	6 (8.3)	10 (11.8)	14 (20.3)	26 (43.3)	
Family Income						0.001
Low	24.5 (7.2)	42 (71.2)	52 (85.3)	40 (62.6)	23 (40.4)	
Intermediate	26.1 (7.1)	13 (22.0)	1 (1.6)	12 (18.7)	16 (28.0)	
High	29.2 (5.9)	4 (6.8)	8 (13.1)	12 (18.7)	18 (31.6)	
City						0.001
Rio de Janeiro	26.3 (7.3)	31 (43.1)	39 (45.3)	41 (59.4)	42 (70.0)	
Manaus	24.1 (7.0)	41 (56.9)	47 (54.7)	28 (40.6)	18 (30.0)	

* Very Weak, 0–21; Weak, 22–27; Intermediate, 28–31; Desirable, 32–47, according to quartiles.
